# Capturing a Patient-Reported Measure of Physical Function Through an Online Electronic Health Record Patient Portal in an Ambulatory Clinic: Implementation Study

**DOI:** 10.2196/medinform.8687

**Published:** 2018-05-09

**Authors:** Jing Li, Jinoos Yazdany, Laura Trupin, Zara Izadi, Milena Gianfrancesco, Sarah Goglin, Gabriela Schmajuk

**Affiliations:** ^1^ Division of Rheumatology Department of Medicine University of California, San Francisco San Francisco, CA United States; ^2^ VA Medical Center San Francisco, CA United States

**Keywords:** electronic health record, patient-reported outcomes, rheumatoid arthritis

## Abstract

**Background:**

Despite significant interest in the collection of patient-reported outcomes to make care more patient-centered, few studies have evaluated implementation efforts to collect patient-reported outcomes from diverse patient populations

**Objective:**

We assessed the collection of patient-reported outcomes from rheumatoid arthritis patients in an academic rheumatology clinic, using a paper and an online form through the electronic health record patient portal.

**Methods:**

We identified patients seen between 2012-2016 with ≥2 face-to-face encounters with a rheumatology provider and International Classification of Diseases codes for RA, ≥30 days apart. In 2013, our clinic implemented a paper version of the Patient Reported Outcome Measurement Information System (PROMIS) physical function form that was administered to patients upon their check-in at the clinic. In 2015, an online version of the form became available by way of the electronic health record patient portal to patients with active portal accounts. We compared the proportion of visits with documented PROMIS scores across age, race and ethnicity, and language and examined trends over time using a control chart.

**Results:**

We included 1078 patients with rheumatoid arthritis with 7049 in-person encounters at the rheumatology clinic over 4 years, with an average of 168 visits per month. Of the included patients, 80.4% of patients (867/1078) were female and the mean age was 58 (SD 16) years. The overall PROMIS physical function score documentation increased from 60.4% (1081/1791) of visits in 2013 to 74.4% (905/1217) of visits in 2016. Online score documentation increased from 10.0% (148/1473) in 2015 to 19.3% (235/1217) in 2016. African American patients were least likely to have a PROMIS physical function score recorded (55/88, 62.5% compared to 792/990, 80.0% for other racial or ethnic groups; *P*<.001). Compared with white patients, both African American and Hispanic patients were less likely to have active online electronic health record portal accounts (44/88, 50% and 90/157, 57.3% respectively, compared to 437/521, 83.9% of white patients; *P*<.001) and, once activated, less likely to use the online survey (6/44, 13.6% and 16/90, 17.8% respectively, compared to 135/437, 30.9% of white patients; *P*=.02). There was no significant difference in the proportion of any PROMIS physical function forms recorded between non-English vs English preferred patients. No significant differences were found across age or gender.

**Conclusions:**

PROMIS physical function form completion improved overall from 2012-2016 but lagged among racial and ethnic minorities and non-English preferred patients. Future studies should address issues of portal access, enrollment, satisfaction, and persistence and focus on developing PRO implementation strategies that accommodate the needs and preferences of diverse populations.

## Introduction

The effective use of patient-reported outcomes (PROs) data is anticipated to play a critical role in improving health care delivery, patient experiences with care, and outcomes. In rheumatoid arthritis (RA), a complex chronic condition characterized by joint pain and inflammation, validated PROs have been used over the past several decades to assess levels of RA disease activity and functional status [1,2]. PROs have successfully informed treatment decisions and facilitated shared decision-making, patient engagement and goal-setting in RA [3-6]. Routine assessment of PROs is now recommended by American College of Rheumatology (ACR) guidelines, and quality measures to encourage the regular collection of RA PROs have been endorsed by the National Quality Forum [7,8]. Despite significant interest in the collection of PROs to make care more patient-centered, few studies have evaluated implementation efforts to collect PROs in real-world practice settings that serve diverse patient populations [9].

Different approaches to collecting RA PROs have been used, including paper questionnaires, telephone interviews, and, more recently, digital health approaches such as electronic health record (EHR) patient portals. Online patient portal-based collection of PROs is appealing in health care settings because, by utilizing the existing infrastructure of the EHR, they have the potential to decrease the burden of data collection and entry for clinic staff and providers. In addition, PRO information collected through the EHR enables tracking of outcomes at the individual patient level over time, a feature that is useful for patients and providers in assessing whether a key treatment goal, maintaining functional capacity, is being achieved.

In this study, we assessed the proportion of RA patients who completed a physical function PRO form prior to an in-person visit, and the fraction of those who used the online EHR portal to report PROs once that functionality was implemented. Because we hypothesized that socio-demographic factors might influence how patients chose to complete PRO surveys, we also examined completion patterns by age, gender, race and ethnicity, and preferred language. Finally, we describe the challenges encountered in implementing RA PROs in our health system.

## Methods

### Clinical Context and Workflows

The University of California, San Francisco (UCSF) is an academic health center with over 750,000 outpatient visits per year that uses an Epic EHR system. The catchment area includes much of northern California. The UCSF rheumatology clinic incorporated physical function assessments into its routine practice February 1, 2013. Workflows were designed such that a paper version of the PROMIS PF survey, the PF-10a, was given to patients upon check in to each of their return visits, with instructions to complete the form in the waiting room prior to their clinical encounter. When patients were called from the waiting room to perform vital signs, medical assistants would calculate the PROMIS score from the paper survey and input the raw score into a flowsheet in the EHR. The raw score is automatically converted into a T-score, a standardized score on a relevant reference population, where 50 is the mean and 10 is the standard deviation (SD) of that population [10].

**Figure 1:**
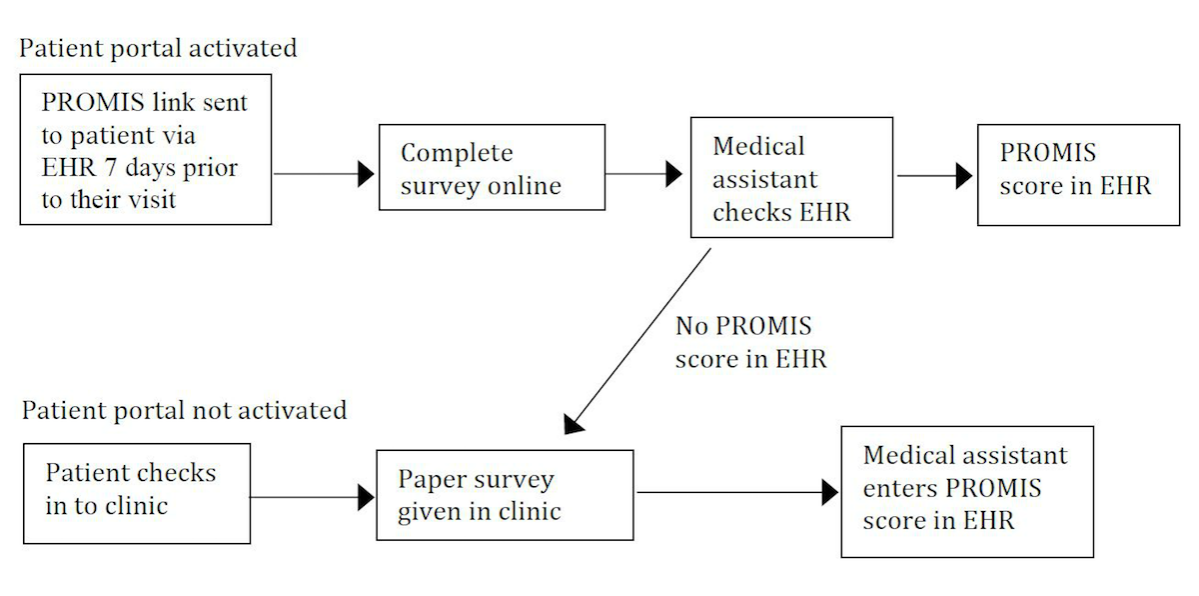
Flow of PROMIS documentation in the clinic. EHR: electronic health record; PROMIS: Patient Reported Outcome Measurement Information System.

Because there was interest in automating PRO measure collection through the EHR, we collaborated with our institutional health information technology specialists to implement PROMIS PF collection through our Epic system’s patient portal, called MyChart. Patients received an activation code at any in-person visit at UCSF that would allow them to log on to the online patient portal. Once the portal was activated, patients received a MyChart message from their provider 7 days prior to their appointment with a link to the PROMIS PF form (PF-12a). This workflow was implemented January 1, 2015 and medical assistants looked for a recorded online PROMIS survey score in the EHR when a patient checked in to the clinic. If a patient had a documented PROMIS PF score through the patient portal within 7 days of their appointment, they would be instructed to skip the paper-based PROMIS PF survey. PROMIS scores collected through the online portal are also automatically converted to T-score and input into a flowsheet within the EHR (Figure 1).

### Patients and Data Source

The UCSF Committee on Human Research approved this study.

Data was derived from our Epic Clarity Data Warehouse and included the denominator of patients who had two or more ICD9 codes (714.0) for RA (at least 30 days apart) between June 1, 2012 and July 31, 2016. Information on the demographics for each patient was extracted (age, gender, self-reported race and ethnicity, and preferred language), online EHR portal activation status as of January 1, 2015 (the date the PROMIS PF became available through the online EHR portal), and, by the end of the study period, rheumatology visit encounter dates, disease activity scores by way of Clinical Disease Activity Index , PROMIS physical function (PF) scores, and PROMIS PF survey completion method (paper or EHR patient portal). Follow-up time was defined as the total number of months between each patient’s first and last visits during the study period.

### PROMIS PF Measures

The PROMIS PF survey was developed by the National Institute of Health [10]. Raw scores range from 10 to 50 and can be transformed into a T-score to compare a given patient’s score to the US general population mean (mean 50, SD 10). In this study, we examined use of the PF-10a (the short form 10-item paper questionnaire), which is available in multiple languages, including English, French, Spanish, and Chinese. In the EHR patient portal, we implemented the PF-12a in English only on January 1, 2015. PF-12a is a short form physical function questionnaire revised from PF-10a. PF-12a raw scores range from 12 to 60 but can be compared directly with PF-10a scores using T-scores [11].

### Primary Analysis

We examined the proportion of patients completing the PROMIS PF-10a after the paper form was implemented in 2013, and the proportion of patients completing the form electronically in 2015 after the online EHR patient portal PRO form was implemented. We used descriptive statistics to summarize age, gender, race and ethnicity, language preference, and online EHR portal activation. This data, and the relationships between patient characteristics and PROMIS PF completion method, mean PROMIS PF score, and mean disease activity score (when available) were examined using analysis of variance (ANOVA) for continuous variables or chi-square tests for categorical variables.

We tested for a potential interaction between age and race and ethnicity in a logistic regression model with portal activation as outcome, categorized age (≥70 and <70), race and ethnicity and cross-product terms as independent variables. In addition, we calculated the proportion of visits per month in which either a paper (PF-10a) or online EHR portal (PF-12a) PROMIS score was recorded in the EHR from 2013 to 2016. The frequencies of PROMIS PF completed by 1) either online EHR portal or paper and 2) the online EHR portal only were plotted monthly on a quality control chart (p-chart) [12]. We calculated the proportion of patients who used and persistently used the online EHR portal to complete PROMIS PF forms. Spearman correlation coefficients were used to test the correlation between the paper and the online EHR portal PROMIS PF T-scores.

### Paper versus Online PROMIS PF Survey Score Correlation

Because some patients were filling out the paper survey, while others were filling out the online EHR portal survey, and still others were switching between methods, we were interested in understanding the correlation of PRO scores when assessed by different means within a short time period. A temporary system error that occurred during a 3-month period in 2016 allowed us to examine this correlation. During this period, due to a bug in a system upgrade, patients’ online EHR portal PROMIS PF scores were not visible to clinic staff at the time of patient check in (N=51 encounters). These patients were asked to complete the paper PROMIS PF in clinic in addition to the online EHR PROMIS PF survey they had already completed. Additionally, there were multiple occasions in which the online EHR portal and paper PROMIS PF were completed within 7 days by the same patient for unclear reasons (N=209 encounters). In the main analysis, paper surveys that were completed within 7 days of online EHR surveys were deleted from the analysis, and only the online EHR portal score was counted (117/1446, 8.1% of paper surveys in 2015; 143/907,15.8% of paper surveys) in 2016). However, these paper and online EHR scores within 7 days of each other provided an opportunity to compare scores across collection methods. When both scores were present, we calculated the proportion of individuals with floor (defined as worst) and ceiling (defined as best) scores for PF-10a and PF-12s and compared these proportions using a *t*-test [10].

Analyses were performed using Stata 14. For all analyses, *P* values <.05 were used as the criterion for statistical significance.

## Results

We included 1078 RA patients with 7049 in-person encounters in the UCSF rheumatology clinic from June 1, 2012 to July 31, 2016. Of the patients, 80.4% (867/1078) were female, with a mean age of 58 years (SD 16; see Table 1). This group was racially and ethnically diverse and 557/1078 (51.7%) identified themselves as non-white, and 150/1078 (13.9%) reported a language other than English as their preferred language, primarily Chinese or Spanish. EHR portal account activation was a required step to complete the online portal PROMIS survey, although most patients who completed exclusively paper surveys (412/627) also had active accounts (65.7%).

**Figure 2:**
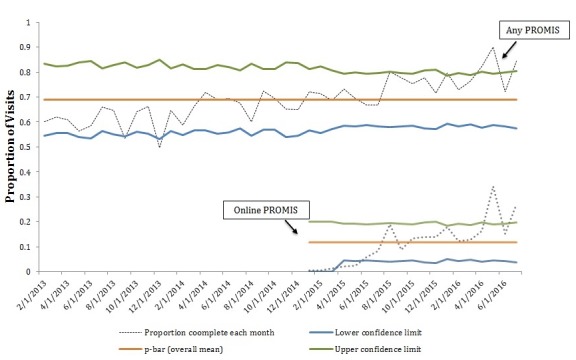
Percent completion of PROMIS by month. Both overall PROMIS and online PROMIS completion are presented. The black dotted line shows the proportion of patient encounters with a documented PROMIS by month. The upper control and low control limits vary as the denominator of patient encounters changed every month. The p-bar (overall mean) shows the average PROMIS documentation rate. More than 6 values are seen above the p-bar over the time, indicating a positive improvement in both overall PROMIS and online PROMIS documentation.

Of all the patients, 78.6% (847/1078) had at least one PROMIS PF score recorded during the study period. There was an average of 168 RA in-person encounters per month over the study period. The proportion of visits with any documented PROMIS PF score increased over time, from 60.4% (1081/1791) in 2013 to 74.4% (905/1217) in 2016. In 2015 (after the online EHR portal PROMIS PF survey became available), 10.0% (148/1473) of visits had an associated online EHR portal PROMIS PF score, rising to 19.3% (235/1217) in 2016 (see Figure 2).

We explored patient factors associated with method of completion of the PROMIS PF (see Table 1). African American patients were less likely to have any PROMIS PF recorded compared with other groups (55/88, 62.5% compared to 792/990 80.0% for other racial and ethnic groups; *P*<.001). Both African American and Hispanic patients were less likely to have active EHR portal accounts compared with white patients (44/88, 50.0% and 90/157, 57.3% 90/157 respectively, compared to 437/521, 83.9% 437/521 of white patients; *P*<.001) and once activated, less likely to have completed the online PROMIS survey (6/44, 13.6% and 16/90, 17.8% respectively, compared to 135/437, 30.9% of white patients; *P*=.02). There was no significant difference in the proportion of any PROMIS PF recorded between non-English vs English preferred patients. However, non-English preferred patients were less likely to have active EHR portal accounts (79/150, 47.3% vs 734/928, 79.1% of English preferred patients; *P*<.001) or to use the online survey (10/71, 14.1% vs 210/734, 28.6% of English preferred patients; *P*=.03), likely because the online survey existed only in English.

Patients who had completed at least one online EHR portal PROMIS PF survey had 3.4 points higher mean PROMIS T-score, although this difference was not statistically significant (42.3 vs 38.9; *P*=.7). Patients who only completed the paper PROMIS PF survey had a significantly higher mean disease activity score (12.9 vs 10.4; *P*<.001). We also found that patients with longer follow-up and more visits per year were more likely to have any PROMIS PF score recorded (*P*<.001). No significant differences in PROMIS completion rate overall or online survey use were found across age or gender.

There were 775 (775/1078, 71.9%) patients who activated their online patient portal, 84.5% of which were activated before January 2015, when the online PROMIS PF survey was available. This rate did not vary by group (paper only, online, no PROMIS). There were no significant differences in patient characteristics when we compared patients who contributed visits before versus after January 2015 (when the online PROMIS survey became available, data not shown). We used multivariate logistic regression to assess the possibility of an interaction between age and race and ethnicity in the patients’ portal activation status. We found that both age and race and ethnicity were associated with significant differences (younger, Caucasian patients were more likely to have active portals, *P*<.001 and *P*<.001, respectively), but that there was no age-race and ethnicity interaction.

Only 220 patients completed the online PROMIS PF survey at least once during the study period. We examined whether patients who completed the online survey once continued to use the online version of the PROMIS survey for future visits. Of these 220 patients, 84 (38.2%) used the online survey intermittently; 112 (112/220, 50.9%) used the online survey only once and reverted to paper surveys thereafter, and the remainder had no additional visits. We found no patients who used the online EHR portal exclusively (after its implementation) over time.

During the 3-month period in which an EHR programming error resulted in clinic staff not being able to view the online EHR portal PROMIS PF scores at the time of patient check-in, 51 patients completed the paper PROMIS PF in the clinic even though they had already completed the online EHR portal version in the days prior to the appointment. We found an additional 157 patients (209 encounters) who completed both paper and online EHR portal scores within 7 days of each other at some point during the study period. This gave us the opportunity to compare paper and online PROMIS scores from the same patients, for the same visit. Among these patients, the online EHR portal PROMIS PF score had a mean T-score of 40.5 (SD 10.7, range 16.1-66.4), and the paper PROMIS PF score had a mean T-score of 43.2 (SD 9.6, range 23.4-61.7), with a Spearman’s correlation of *r*=0.68 (*P*<.001). The paper PROMIS PF had a significant 2.7 point higher mean T-score by paired *t*-test (*P*<.001).

**Table 1:** Patient characteristics by PROMIS survey completion methods, n (%).

		All Patients (n=1078)	Paper Survey Only (n=627)	At Least 1 Online EHR^a^ Portal Survey (n=220)	No PROMIS^b^ Survey Completed (n=231)	*P* value^c^
Age (years), mean (SD)		58 (16)	58 (16)	56 (15)	58 (17)	.7
**Gender, n (%)**						**.4**
	Female	867 (80.4)	508 (81.0)	181 (82.3)	178 (77.1)	
	Male	211 (19.6)	119 (19.0)	39 (17.7)	53 (22.9)	
**Race or Ethnicity, n (%)**						**<.001**
	White	521 (48.3)	281 (44.8)	135 (61.4)	105 (45.4)	
	African American	88 (8.2)	49 (7.8)	6 (2.7)	33 (14.3)	
	Asian	149 (13.8)	103 (16.4)	26 (11.8)	20 (8.7)	
	Hispanic	157 (14.6)	105 (16.8)	16 (7.3)	36 (15.6)	
	Other or Multiple	163 (15.1)	89 (14.2)	37 (16.8)	37 (16)	
**Preferred Language, n (%)**						**<.001**
	English	928 (86.1)	519 (82.8)	210 (95.4)	199 (86.2)	
	Other^d^	150 (13.9)	108 (17.2)	10 (4.6)	32 (13.8)	
Online EHRs portal activated prior to January 2015, n (%)		655 (60.8)	343 (54.7)	189 (85.9)	123 (53.2)	<.001
Online EHR portal activated prior to end of study period, n (%)		775 (71.9)	412 (65.7)	220 (100.0)	143 (61.9)	<.001
Visits per patient, per year, mean (SD)		3.8 (2.0)	4.0 (1.9)	4.5 (1.8)	2.4 (2.0)	<.001
Follow-up months, mean (SD)		23 (0.5)	24 (0.6)	30 (0.8)	10 (1)	.05
Disease activity score (CDAI)^e^, mean (SD)		12.0 (11.0)	12.9 (11.4)	10.4 (10.1)	19.4 (3.2)	<.001
PROMIS scores, mean (SD)		40.1 (10.8)	38.9 (11.1)	42.3 (10.0)^f^	N/A^g^	.7

^a^EHR: electronic health record.

^b^PROMIS: Patient Reported Outcome Measurement Information System.

^c^*P* values were tested by ANOVA tests for continuous variables or chi-square for categorical variables.

^d^Other included unknown or declined, n=5.

^e^681 patients with Clinical Disease Activity Index (CDAI) recorded: 480 paper survey only; 198 at least one online survey; 3 no survey completed.

^f^260 paper records were excluded: 209 PROMIS Physical Function scores because of duplicate online score from the same visit; 51 scores because of the temporary systematic error.

^g^Not applicable.

## Discussion

To our knowledge, this is the first study to describe utilization of an online EHR patient portal for the collection of PROs as part of the routine care of a population of patients with RA. We found that over a 4-year period, overall PROMIS PF survey completion increased by 23.2%. Still, implementation was suboptimal, with more than 20.0% of in-person encounters lacking a PROMIS PF score. PROMIS measurement lagged particularly among racial and ethnic minorities. The proportion of patients completing PROMIS PF surveys through the online EHR portal almost doubled from the time of its implementation in 2015, yet only accounted for only 19.3% of the all PROMIS PF measurements. We found no persistence in the use of the online EHR portal for PROMIS reporting over time.

Few studies have described PRO collection via an online portal. One study in an ambulatory cancer care setting described a very successful implementation of electronic collection of PROs to measure common cancer symptoms (including physical function). PROs were measured by way of an online portal survey prior to clinical visits and were found to be an effective basis for referral for psychosocial and supportive care [13]. However, this study included only patients who activated and enrolled in the online portal and did not provide information on patients unable or unwilling to do so.

Our study found that most patients had an active online portal account before the PROMIS PF survey was available by way of the online EHR portal in January 2015, although only a small fraction completed the online EHR portal PROMIS survey, and that use lagged among racial and ethnic minorities. There are multiple possible reasons for this difference by race and ethnicity. First, there may be a lack of support and training to assist and encourage patients with portal activation, which is crucial to the successful implementation of an online measurement system [14]. Our findings are consistent with prior studies showing that racial and ethnic minorities are generally less likely to enroll and utilize online EHR portals [15-18]. Second, qualitative studies exploring possible reasons for reduced use of online EHR portals among African American and Hispanic patients have highlighted technical barriers and worries that use of a portal could undermine in-person relationships with healthcare providers [19]. Third, language proficiency may have hindered online EHR portal access—only 14.1% of patients with a preferred language other than English completed the online survey, compared to 28.6% of patients preferring English. Providing materials including enrollment information, websites and surveys, in other languages and for low literacy patients will be an important advance for increasing online EHR portal use [20,21]. Interestingly, although prior studies have reported that older adults from racial and ethnic minorities are significantly less likely to access and use an online patient portal [22], we found no evidence of a significant interaction between age and race and ethnicity on online EHR portal activation use in our study.

We found that patients who had completed at least one online survey seemed to have a lower disease burden compared to patients who completed only paper surveys (as evidenced by marginally better functional status and lower disease activity scores). Although this study wasn’t designed to study why this was the case, we hypothesize that completing the online survey may be more burdensome or difficult for patients with more active disease. Future qualitative studies could investigate this further.

Perhaps of greatest concern, our study found that no patients used the online EHR portal consistently for PRO measure collection over time; over half of patients abandoned the online portal for PRO reporting after a single use. To our knowledge, this lack of persistence in use has not been reported previously. Reasons for this could be multiple, including the lack of integration into routine care progress or dissatisfaction with the online PRO collection process or time constraints. Disruptions in the EHR that resulted in clinic staff not being able to access online PRO scores due to a system error, necessitating double entry of PRO scores, may have frustrated patients and caused them to abandon online EHR portal use. Future work on online EHR portals should focus both on barriers to enrollment and barriers to persistence in use.

In our comparison of paper and online PROMIS PF T-scores for the same patient and same visit, we found moderate levels of correlation between the PF-10a (paper) and PF-12a (online) versions *(r*=0.68; *P*<.001). Although it is valid to compare results of these 2 scores [10], the small difference that we detected is likely due to a difference in psychometric properties of the PF-10a and the PF-12a. Specifically, there was a significant difference in ceiling effects between the two versions, with the paper version having a higher ceiling effect (8.9% vs 0%; *P*<.001). No floor effects were observed.

The limitations of this study include a lack of information about patients’ internet and computer access, and lack of a measure of education or healthy literacy level. Studies have shown that patients with low health literacy experience basic technological barriers such as difficulty using a mouse or finding specific keys on the keyboard in addition to “routine” technological barriers such as mistyping and navigation issues [23]. Future assessments should aim to capture this important variable.

Our study shows that the potential benefits of online EHR portal collection of PROs have not yet been realized in the UCSF rheumatology clinic. One goal of online collection was to decrease the burden of data collection and data entry. In our case, the clinic workflow still required that medical assistants assess whether a patient already completed an online version of the survey at the time of patient check-in. This process was both time-consuming and faulty (as evidenced by the numerous patients who completed both online and paper surveys within 7 days of an in-person visit). Another goal of online PRO collection was to enable automated tracking of outcomes over time. However, 1.5 years after implementation, uptake of the online EHR portal is poor at only 19.3% of visits. Further study is needed to investigate and address these issues, and pragmatic trials should test strategies to optimize the collection of PROs by addressing patient, provider, and health-system factors.

In summary, despite increasing completion of PROMIS PF surveys, we found only a fraction of patients who were offered online EHR portal use completed their PROMIS PF survey online, and none used it persistently. Disparities exist across race and ethnicity and language in access to the online EHR portal and in PRO completion once the portal is activated. Future studies should address issues of portal access, enrollment, satisfaction and persistence, and focus on developing PRO implementation strategies that accommodate the needs and preferences of diverse populations.

## Abbreviations

ACR: American College of Rheumatology
ANOVA: analysis of variance
EHR: electronic health record
PF: physical function
PROMIS: Patient Reported Outcome Measurement Information System
PRO: patient-reported outcome
RA: rheumatoid arthritis

## References

[1] Boers M, Tugwell P, Felson DT, van Riel PL, Kirwan JR, Edmonds JP, Smolen JS, Khaltaev N, Muirden KD (1994). World Health Organization and International League of Associations for Rheumatology core endpoints for symptom modifying antirheumatic drugs in rheumatoid arthritis clinical trials. J Rheumatol Suppl.

[2] van Tuyl LHD, Boers M (2015). Patient-reported outcomes in core domain sets for rheumatic diseases. Nat Rev Rheumatol.

[3] Malysheva O, Bedrich A, Kuipers JG, Kleine H, Wolff B, Baerwald CG (2015). Use of clinical scores to guide therapeutic decisions in patients with rheumatoid arthritis in daily care. Clin Exp Rheumatol.

[4] Ovretveit J, Keller C, Hvitfeldt FH, Essén A, Lindblad S, Brommels M (2013). Continuous innovation: developing and using a clinical database with new technology for patient-centred care--the case of the Swedish quality register for arthritis. Int J Qual Health Care.

[5] Newman ED, Lerch V, Billet J, Berger A, Kirchner HL (2015). Improving the quality of care of patients with rheumatic disease using patient-centric electronic redesign software. Arthritis Care Res (Hoboken).

[6] Lavallee DC, Chenok KE, Love RM, Petersen C, Holve E, Segal CD, Franklin PD (2016). Incorporating Patient-Reported Outcomes Into Health Care To Engage Patients And Enhance Care. Health Aff (Millwood).

[7] Singh JA, Saag KG, Bridges SL, Akl EA, Bannuru RR, Sullivan MC, Vaysbrot E, McNaughton C, Osani M, Shmerling RH, Curtis JR, Furst DE, Parks D, Kavanaugh A, O'Dell J, King C, Leong A, Matteson EL, Schousboe JT, Drevlow B, Ginsberg S, Grober J, St CEW, Tindall E, Miller AS, McAlindon T, American College of Rheumatology (2016). 2015 American College of Rheumatology Guideline for the Treatment of Rheumatoid Arthritis. Arthritis Care Res (Hoboken).

[8] National Quality Forum NQF-Endorsed measures for musculoskeletal conditions.

[9] Harle CA, Lipori G, Hurley RW (2016). Collecting, Integrating, and Disseminating Patient-Reported Outcomes for Research in a Learning Healthcare System. EGEMS (Wash DC).

[10] Wahl E, Gross A, Chernitskiy V, Trupin L, Gensler L, Chaganti K, Michaud K, Katz P, Yazdany J (2017). Validity and Responsiveness of a 10-Item Patient-Reported Measure of Physical Function in a Rheumatoid Arthritis Clinic Population. Arthritis Care Res (Hoboken).

[11] Oude Voshaar MAH, ten Klooster PM, Glas CAW, Vonkeman HE, Krishnan E, van de Laar MAFJ (2014). Relative performance of commonly used physical function questionnaires in rheumatoid arthritis and a patient-reported outcomes measurement information system computerized adaptive test. Arthritis Rheumatol.

[12] Duclos A, Voirin N (2010). The p-control chart: a tool for care improvement. Int J Qual Health Care.

[13] Wagner LI, Schink J, Bass M, Patel S, Diaz MV, Rothrock N, Pearman T, Gershon R, Penedo FJ, Rosen S, Cella D (2015). Bringing PROMIS to practice: brief and precise symptom screening in ambulatory cancer care. Cancer.

[14] van der Vaart R, Drossaert CHC, Taal E, Drossaers-Bakker KW, Vonkeman HE, van de Laar MAFJ (2014). Impact of patient-accessible electronic medical records in rheumatology: use, satisfaction and effects on empowerment among patients. BMC Musculoskelet Disord.

[15] Sarkar U, Karter AJ, Liu JY, Adler NE, Nguyen R, López A, Schillinger D (2011). Social disparities in internet patient portal use in diabetes: evidence that the digital divide extends beyond access. J Am Med Inform Assoc.

[16] Lyles CR, Harris LT, Jordan L, Grothaus L, Wehnes L, Reid RJ, Ralston JD (2012). Patient race/ethnicity and shared medical record use among diabetes patients. Med Care.

[17] Garrido T, Kanter M, Meng D, Turley M, Wang J, Sue V, Scott L (2015). Race/ethnicity, personal health record access, and quality of care. Am J Manag Care.

[18] Goel MS, Brown TL, Williams A, Hasnain-Wynia R, Thompson JA, Baker DW (2011). Disparities in enrollment and use of an electronic patient portal. J Gen Intern Med.

[19] Lyles CR, Allen JY, Poole D, Tieu L, Kanter MH, Garrido T (2016). “I Want to Keep the Personal Relationship With My Doctor”: Understanding Barriers to Portal Use among African Americans and Latinos. J Med Internet Res.

[20] Bates DW, Bitton A (2010). The future of health information technology in the patient-centered medical home. Health Aff (Millwood).

[21] Luque AE, van Kenken A, Winters P, Keefer MC, Sanders M, Fiscella K (2013). Barriers and Facilitators of Online Patient Portals to Personal Health Records Among Persons Living With HIV: Formative Research. JMIR Res Protoc.

[22] Smith SG, O'Conor R, Aitken W, Curtis LM, Wolf MS, Goel MS (2015). Disparities in registration and use of an online patient portal among older adults: findings from the LitCog cohort. J Am Med Inform Assoc.

[23] Tieu L, Sarkar U, Schillinger D, Ralston JD, Ratanawongsa N, Pasick R, Lyles CR (2015). Barriers and Facilitators to Online Portal Use Among Patients and Caregivers in a Safety Net Health Care System: A Qualitative Study. J Med Internet Res.

